# Successful Implementation and Development of a Phase II Cardiac Rehabilitation Program: A China-Wide Cross-Sectional Study Tracking In-service Training Clinical Staff

**DOI:** 10.3389/fpubh.2021.639273

**Published:** 2021-03-17

**Authors:** Xun Gong, Wenliang Zhang, Jeffrey W. Ripley-Gonzalez, Yuan Liu, Yaoshan Dun, Fan Zheng, Ling Qiu, Suixin Liu

**Affiliations:** ^1^Division of Cardiac Rehabilitation, Department of Physical Medicine and Rehabilitation, Xiangya Hospital of Central South University, Changsha, China; ^2^National Clinical Research Center for Geriatric Disorders, Xiangya Hospital of Central South University, Changsha, China; ^3^Division of Preventive Cardiology, Department of Cardiovascular Medicine, Mayo Clinic, Rochester, MN, United States

**Keywords:** cardiac rehabilitation, implementation and development, independent predictors, quality, survey

## Abstract

**Background:** Despite the benefits of cardiac rehabilitation (CR), phase II CR remains highly unavailable; the factors influential to the successful implementation and development of phase II CR programs have not been fully explored.

**Methods:** A cross-sectional survey was completed by 168 nationwide clinical staff. Parameters associated with the successful implementation and development of phase II CR and the factors associated with the quality of CR were explored by multivariable logistic regression.

**Results:** One hundred and eighteen of 168 respondents' institutions had successfully developed phase II CR programs, 41 of which delivered high-quality CR. Independent factors associated with successful implementation and development of CR were leadership support from hospital administrators, support from resident physicians, staff perception in CR increasing medical risk, and department type (cardiology vs. rehabilitation department). Independent factors associated with CR quality were the availability of “professional CR providers” and staff perceptions of CR improving physician–patient relationships. The medical system factors did not affect the development and quality of CR, including hospital level, funding type, academic type, general/specialized hospital, located city, medical insurance, the existence of a CR outpatient clinic and independent space, the availability of professional CR providers, staff structure, and the availability of regular training and standard procedure.

**Conclusions:** The development and quality of a phase II CR program may benefit from factors including support from administrators and resident physicians, adequately training more CR providers, without viewing medical system factors as a major issue.

## Introduction

Cardiovascular diseases (CVDs) are the leading cause of death worldwide (1, 2). Cardiac rehabilitation (CR) can aid in reducing morbidity and all-cause mortality, including from CVDs, and is recommended as 1A class by multinational guidelines (2, 3). CR is composed of three distinct phases. Phase II of CR offers services to patients following an acute cardiac event or hospitalization and plays a pivotal role in the whole process of CVDs treatment and rehabilitation. However, phase II CR remains highly unavailable, even in recent years. CR programs are currently carried out in ~40% of countries worldwide (4) and only 22.1% in low- and middle-income countries (5). Therefore, exploring the factors in the implementation and development of CR is conducive to accelerating its development.

To understand the barriers of phase II CR development and promote the development of CR, previous studies have been carried out on the barriers encountered by CR both in China and abroad. Studies abroad have investigated the barriers of CR delivery in already developed programs mainly focusing on exploring the factors affecting the referral rate, admission rate, and compliance of patients (6–10). These factors are multifactorial, including patient factors, those of healthcare providers, and medical system levels (9, 11–15). Few studies from China have briefly explored these issues. Although Wang et al. (16) had reported that medical system factors are a major obstacle to the development of CR programs, this study included a small sample of 18 medical staff from a single center. The above-mentioned research mainly discussed these issues in CR from the perspective of patients, CR procedure, and medical insurance policy, but there is no research focusing on CR providers themselves. In addition, there is still a lack of relevant research on the factors that determine CR quality.

China has the largest population with cardiovascular diseases; even so, there were only 216 medical institutions in China developing CR programs until 2018 (17), and many other institutions are preparing for implementation since then. At present, the development of CR programs in China is in its infancy, and the barriers encountered may be representative and of reference significance, especially for low- and middle-income developing countries. In this study, we conducted a nationwide cross-sectional study using an online questionnaire survey of in-service training medical staff. Then, we conducted the investigation and analysis of the influencing factors of phase II of CR implementation and development and further explored the factors related to the quality of CR to provide evidence for the successful implementation and development of phase II CR in China and the undeveloped districts.

## Materials and Methods

### Design and Procedure

A national cross-sectional design was used in the study. The investigation conforms with the principles outlined in the Declaration of Helsinki and was approved by the Medical Ethics Committee of Xiangya Hospital of Central South University (Ethics number: 202007212). In-service training clinical staff trained in the CR Center of Xiangya Hospital were contacted through telephone or messages. The online survey was sent via WeChat (a messaging/calling app) to participants with informed consent. The survey was collected from July to September 2019.

### Sample

Inclusion criteria were the following: (1) in-service training clinical staff who completed the CR training in the CR Center of Xiangya Hospital; (2) 3–6 months training length; (3) undertook training between January 2013 and December 2018; (4) Passed the CR certificate exam; and (5) offered accurate contact information.

### Measures

The following steps were taken to design the survey (18): (1) literature review (databases including MEDLINE, EMBASE, Google Scholar, CNKI, Wanfang Data) to identify studies reporting results of CR program surveys on a regional, national, or greater basis; (2) invited three cardiac rehabilitation specialists and eight in-service training clinical staff created a 30-item survey. The survey comprised four parts, including characteristics of in-service training clinical staff and their affiliated institutions; knowledge, perceptions, and attitudes toward CR; availability and characteristics of CR programs (19), space, equipment, and management system and human resources for CR (a version of the questionnaire translated to English is described in the Supplementary Material Methods and Survey 1).

### Standards for the Development of Phase II CR Program

Phase II CR programs that offered services to patients following an acute cardiac event or hospitalization were of interest. The criteria of phase II CR programs were the following: (1) initial assessment or risk assessment/stratification; (2) structured exercise training program (supervised or not); and (3) at least one other strategy to control CV risk factors (20).

### Overall Quality of CR

Overall, 16 structure and process quality indicators were assessed to evaluate the quality of CR through the survey (20). Eight core components (structure indicators) were recorded, including initial assessment, risk assessment/stratification, exercise training, patient education, management of cardiovascular (CV) risk factors, nutrition counseling, stress management, and tobacco cessation interventions. Eight risk factors (process indicators) interventions were recorded, including blood pressure, lipids, physical inactivity, poor diet, adiposity, tobacco use, glucose/HbA1c, and depression. The CR programs providing 16 quality indicators were categorized as high-quality CR, otherwise lower quality (21, 22).

### Data Analysis

**Figure 1:**
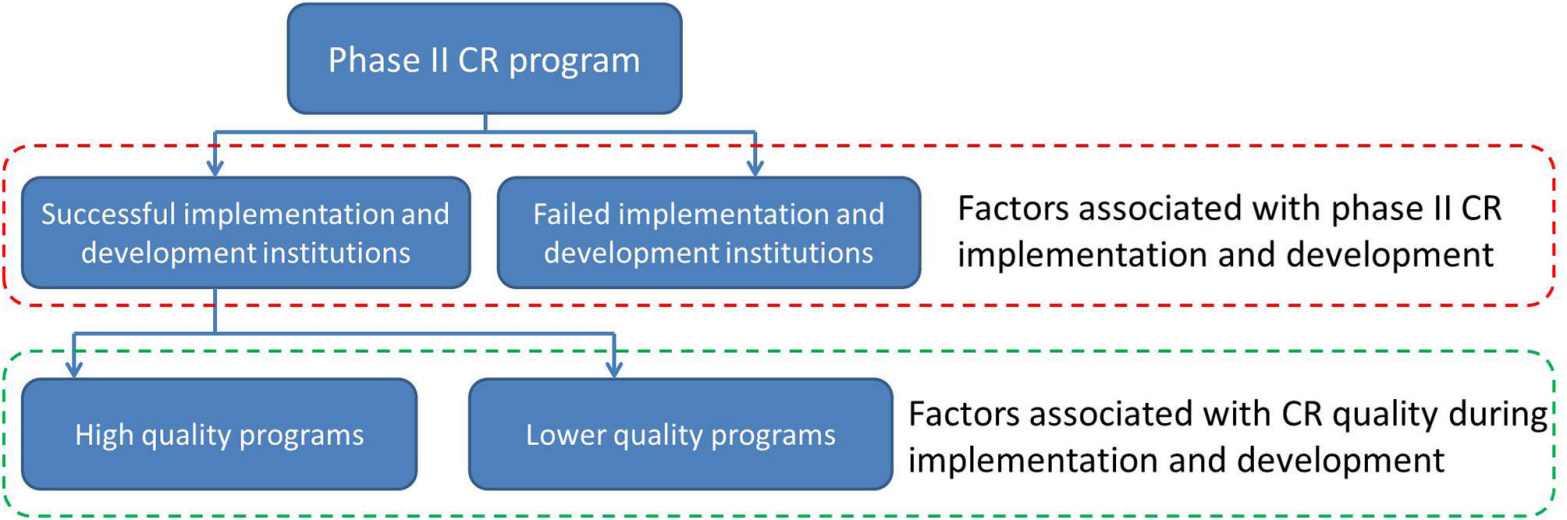
Data analysis flow chart.

Data analysis was performed with SPSS (version 24 for Windows; IBM Corp, Armonk, New York, USA). Data are described in frequency (percentage) or mean (SD). To explore the factors associated with CR development, all cases were divided into two groups (successful implementation and development institutions, failed implementation and development institutions). Furthermore, to explore the factors associated with CR quality during implementation and development, all cases of successful implementation and development institutions were divided into either a high- or a lower-quality group (Figure 1). A chi-square test was used to compare group differences in categorical variables, and an independent-sample *t* test was used for quantitative variables. The factors with *P* < 0.05 were chosen for the next multivariable logistic regression analysis; *P* < 0.05 was considered statistically significant. The odds ratio (OR) and 95% confidence interval (95% CI) were also calculated. (More detailed information of “Methods” are described in Supplementary Material Methods and Survey 1).

## Results

### General Characteristics of the Subjects

**Figure 2:**
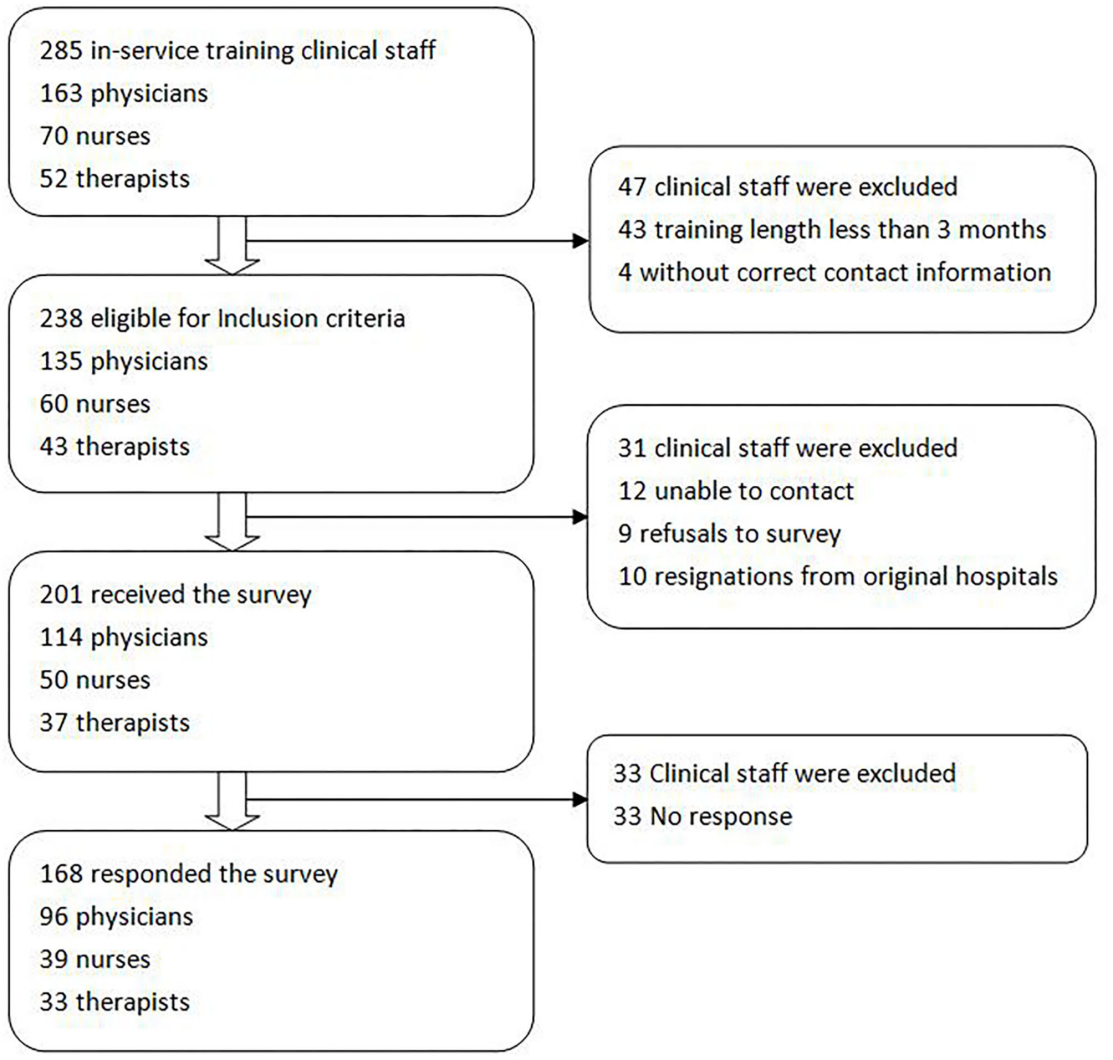
Flow chart of the survey.

This study enrolled 238 in-service clinical staff and received 168 responses, a response rate of 70.6% (Figure 2). One hundred and eighteen (70.2%) respondents' affiliated institutions have already developed phase II CR program.

### Factors Associated With Phase II CR Implementation and Development

#### Characteristics of Respondents and Their Affiliated Institutions

The rate of successful CR implementation and development was higher in cardiology departments than in rehabilitation departments. Hospitals in China are designated as Primary, Secondary, or Tertiary institutions (23). The cities in which affiliated institutions are located were regrouped into five classes using the latest population-based city classifications released by the Chinese State Council (24). The located city of respondents covered 28 provinces and autonomous regions and municipalities, which consisted of 90.3% of administrative areas in mainland China. Overall, most of these characteristics showed no difference between the successful and failed implementation and development institutions, including age, academic degree, clinical title, job title, affiliated institution's level, funding type, general/specialized, academic type, and located city level (Table 1).

**Table 1:** Factors associated with phase II CR implementation and development.

		**Total** ** (*n* = 168)**	**Successful implementation and development institutions** ** (*n* = 118)**	**Failed implementation and development institutions** ** (*n* = 50)**	***P*-value**
**Characteristics of respondents and affiliated medical institutions**					
**Respondents**					
Gender	Female	126(75.0)	94(79.7)	32(64.0)	**0.03**
	Male	42(25.0)	24(20.3)	18(36.0)	
Age, year, mean(SD)		36(7)	37(6)	36(7)	0.58
Educational level	Junior college	9(5.4)	6(5.1)	3(6.0)	0.87
	Bachelor	104(61.9)	72(61.0)	32(64.0)	
	Master and above	55(32.7)	40(33.9)	15(30.0)	
Clinical title	Physician	96(57.1)	66(55.9)	30(60.0)	0.30
	Nurse	39(23.2)	31(26.3)	8(16.0)	
	Therapist	33(19.6)	21(17.8)	12(24.0)	
Job title	Senior	13(7.7)	8(6.8)	5(10.0)	0.20
	Subsenior	31(18.5)	26(22.0)	5(10.0)	
	Intermediate	79(47.0)	56(47.5)	23(46.0)	
	Primary	45(26.8)	28(23.7)	17(34.0)	
Department	Cardiology	126(75.0)	98(83.1)	28(56.0)	** <0.001**
	Rehabilitation	42(25.0)	20(16.9)	22(44.0)	
**Affiliated institution**					
Level	Secondary	33(19.6)	25(21.2)	8(16.0)	0.47
	Tertiary	135(80.4)	93(78.8)	42(84.0)	
Funding type	Public	159(9.6)	114(96.6)	45(90.0)	0.17
	Private	9(5.4)	4(3.4)	5(10.0)	
Category	General	140(83.3)	99(83.9)	41(82.0)	0.76
	Specialized	28(16.7)	19(16.1)	9(18.0)	
Academic type	Teaching Hospital	72(42.9)	50(42.4)	22(44.0)	0.85
	Non-teaching Hospital	96(57.1)	68(57.6)	28(56.0)	
Level of cities	Mega-city I	34(20.2)	25(21.2)	9(18.0)	0.16
	Mega-city II	56(33.3)	42(35.6)	14(28.0)	
	Large city I	38(22.6)	22(18.6)	16(32.0)	
	Large city II	20(11.9)	12(10.2)	8(16.0)	
	Medium- and small-sized city^*^	20(11.9)	17(14.4)	3(6.0)	
**Knowledge and perceptions of CR**					
**Benefits and advantages of CR**					
Reduction in the morbidity and mortality of cardiovascular events		160(95.2)	114(96.6)	46(92.0)	0.38
Improvement of quality of life		161(95.8)	116(98.3)	45(90.0)	**0.04**
Improvement of mental health		152(90.5)	111(94.1)	41(82.0)	**0.03**
Helping patients to return to home and society		146(86.9)	105(89.0)	41(82.0)	0.22
Improvement of physician–patient relationships		119(70.8)	86(72.9)	33(66.0)	0.37
Improvement of the clinical staff's specialized knowledge		125(74.4)	86(72.9)	39(78.0)	0.49
Increased income of clinical staffs		46(27.4)	30(25.4)	16(32.0)	0.38
**Disadvantages of CR**					
Increased medical risk		89(53.0)	56(47.5)	33(66.0)	**0.03**
Greatly increased workloads		85(50.6)	62(52.5)	23(46.0)	0.44
Clinical staff's wages not greatly increased		85(50.6)	64(54.2)	21(42.0)	0.15
**Attitudes toward CR (whether supportive of CR)**					
Resident physicians		144(85.7)	108(91.5)	36(72.0)	**0.001**
Senior physicians		153(91.1)	111(94.1)	42(84.0)	0.07
Nurses or therapists		148(88.1)	107(90.7)	41(82.0)	0.11
Head nurses or head therapists		155(92.3)	112(94.9)	43(86.0)	0.10
Department administrators		159(94.6)	116(98.3)	43(86.0)	**0.004**
Hospital administrators		145(86.3)	110(93.2)	35(70.0)	** <0.001**

* *Mega-city I (>10 million inhabitants), Mega-city II (between 5 and 10 million inhabitants), Large city I (between 3 and 5 million inhabitants), Large city II (between 1 million and 3 million inhabitants). Medium- and small-sized city (<1 million inhabitants)*.

*ata are presented as number (percentage) unless otherwise indicated*.

*CR, cardiac rehabilitation. Bold values indicate that the value is p < 0.05*.

#### Clinical Staff's Knowledge, Perceptions, and Attitudes Toward CR

Concerning the benefits of CR, the successful implementation and development of phase II CR group's staff were more optimistic about “Improvement of quality of life” (98.3 vs. 90.0%, *P* = 0.04) and “Improvement of mental health” (94.1 vs. 82.0%, *P* = 0.03). Fewer of those from the successful implementation and development of phase II CR group considered “Increased medical risk” (47.5 vs. 66.0%, *P* = 0.03) a disadvantage. All clinical staff professionals were largely supportive of CR (≥85.7%). Resident physicians, department administrators, and hospital administrators showed more positive attitudes toward CR in successful implementation and development of phase II CR group than those in unsuccessful implementation and development of phase II CR group (91.5 vs. 72.0%; 98.3 vs. 86.0%; 93.2 vs. 70.0%, respectively) (Table 1).

### Factors Associated With Phase II CR Implementation and Development

**Figure 3:**
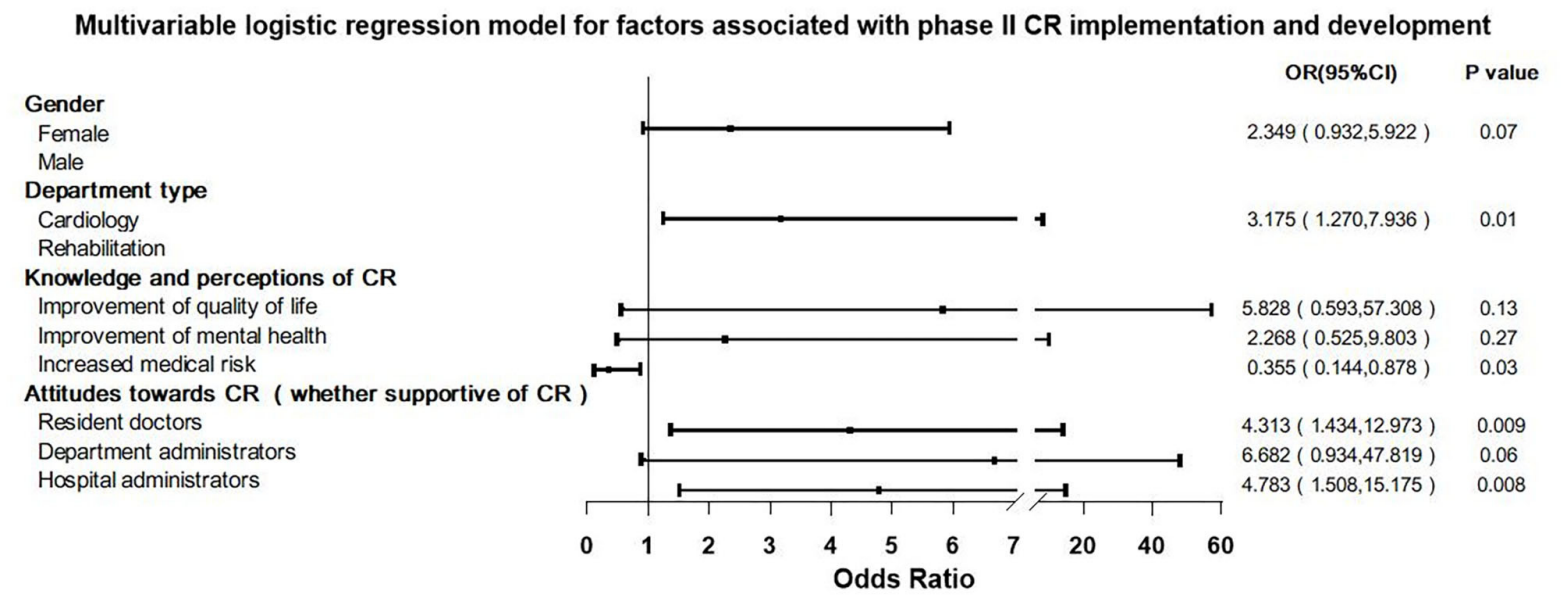
Multivariable logistic regression model for factors associated with phase II cardiac rehabilitation (CR) implementation and development.

Multivariable logistic regression showed that “Department type (cardiology vs. rehabilitation department),” “Resident physicians support for CR,” and “Hospital administrators support for CR” are positive independent predictors, and their OR (95% CI) values were 3.175 (1.270, 7.936), 4.313 (1.434, 12.973), and 4.783 (1.508, 15.175), respectively. “Clinical staff perception that there is increased medical risk in CR” was a negative predictor and its OR value was 0.355 (0.144, 0.878) (Figure 3).

### General Characteristics of Phase II CR Quality During Implementation and Development

**Figure 4:**
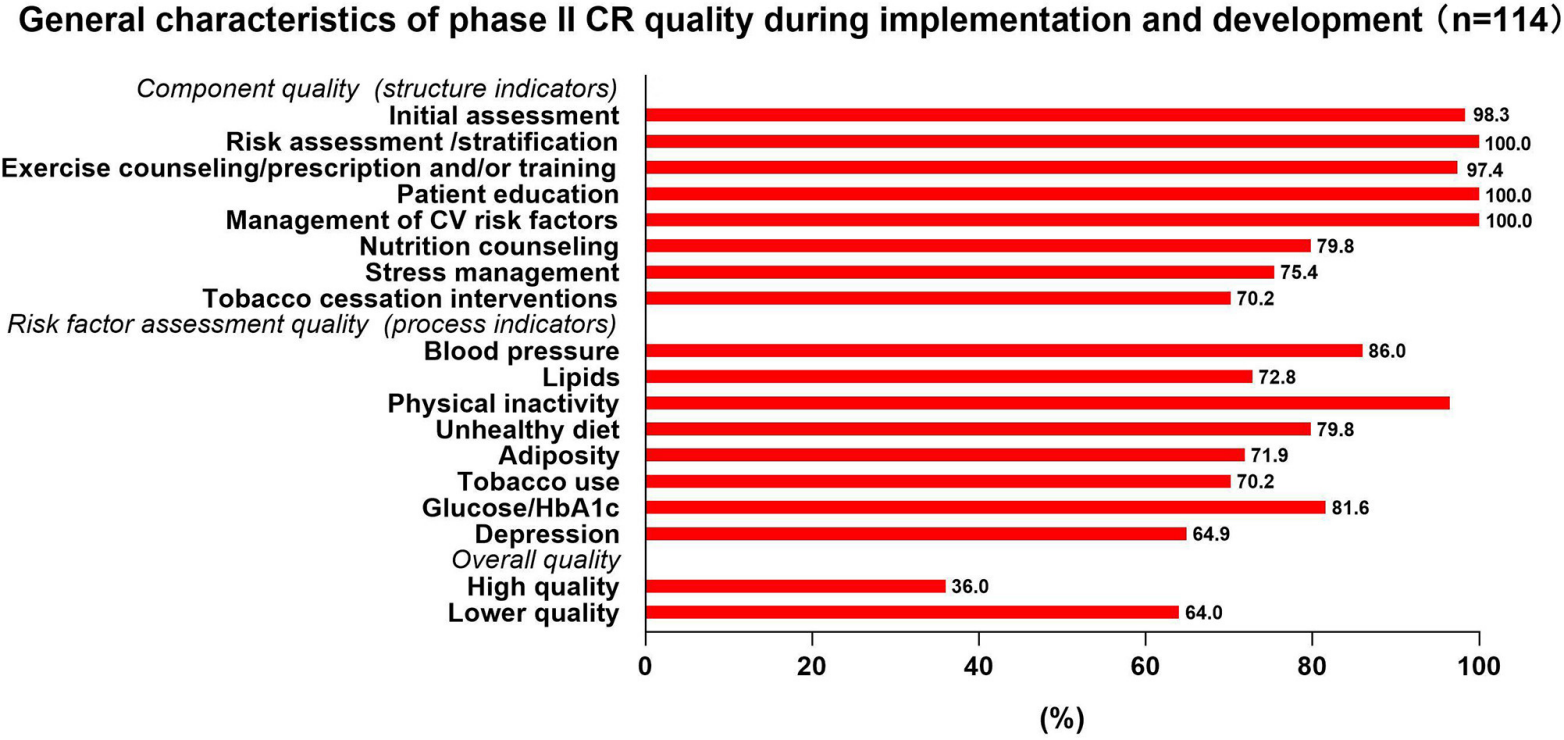
General characteristics of phase II cardiac rehabilitation (CR) quality during implementation and development.

The study showed that 118 (70.2%) affiliated institutions had already developed phase II CR programs, 114 provided 16 indictors about CR quality, and 41/114 (36.0%) of institutions delivered high-quality CR (Figure 4).

### Medical System Factors Associated With Phase II CR Quality During Implementation and Development

There were significant differences in independent space, the availability of professional CR providers, regular CR training, and standard procedure between the two groups. Regarding staff structure in the CR team, there were differences in the composition of nurses and therapists between two groups, and a higher proportion of “both nurses and therapists” and “either nurses or therapists” (compared with “no nurses or therapists”) were observed in the high-quality group (Table 2).

**Table 2:** Factors associated with phase II CR quality during implementation and development.

		**Total** ** (*n* = 114)**	**High quality group** ** (*n* = 41)**	**Lower quality group** ** (*n* = 73)**	***P*-value**
**Medical system factors associated with phase II CR quality**					
**Characteristics of affiliated institutions**					
Level	II	24(21.1)	9(22.0)	15(20.5)	0.86
	III	90(78.9)	32(78.0)	58(79.5)	
Funding type	Public	110(96.5)	39(95.1)	71(97.3)	0.95
	Private	4(3.5)	2(4.9)	2(2.7)	
Category	General	95(83.3)	35(85.4)	60(82.2)	0.66
	Specialized	19(16.7)	6(14.6)	13(17.8)	
Academic type	Teaching Hospital	49(43.0)	20(48.8)	29(39.7)	0.35
	Non-teaching Hospital	65(57.0)	21(51.2)	44(60.3)	
Level of cities	Mega-city I	23(20.2)	6(14.6)	17(23.3)	0.22
	Mega-city II	41(36.0)	17(41.5)	24(32.9)	
	Large city I	22(19.3)	6(14.6)	16(21.9)	
	Large city II	12(10.5)	3(7.3)	9(12.3)	
	Medium- and small-sized city	16(14.0)	9(22.0)	7(9.6)	
Department type	Cardiology	94(82.5)	35(85.4)	59(80.8)	0.54
	Rehabilitation	20(17.5)	6(14.6)	14(19.2)	
**Space, equipment, management system, and human resources**					
CR outpatient clinic		39(34.2)	15(36.6)	24(32.9)	0.69
Independent space		88(77.2)	36(87.8)	52(71.2)	0.04
Professional CR providers		76(66.7)	34(82.9)	42(57.5)	0.006
**Structure of CR team**					
Physicians		103(90.4)	40(97.6)	63(86.3)	0.10
Nurses and therapists					
Both		49(43.0)	24(58.6)	25(34.2)	0.03
Either		58(50.9)	16(39.0)	42(57.6)	
None		7(6.1)	1(2.4)	6(8.2)	
Regular CR training		78(68.4)	34(82.9)	44(60.3)	0.01
Standard procedure		82(71.9)	36(87.8)	46(63.0)	0.005
**Medical insurance**					
Coverage		111(97.4)	40(97.6)	71(97.3)	1.00
Reimbursement rate					
75.0–100.0%		19(16.7)	9(22.0)	10(13.7)	0.31
50.0–74.9%		41(36.0)	12(29.3)	29(39.7)	
25.0–49.9%		29(25.4)	13(31.7)	16(21.9)	
0.0–24.9%		25(21.9)	7(17.1)	18(24.7)	
**Clinical staff factors associated with CR quality**					
**Knowledge and perceptions of CR**					
**Benefits and advantages of CR**					
Reduction in the morbidity and mortality of cardiovascular events		110(96.5)	41(100.0)	69(94.5)	0.32
Improvement of quality of life		112(98.2)	41(100.0)	71(97.3)	0.54
Improvement of mental health		107(93.9)	41(100.0)	66(90.4)	0.10
Helping patients to return to home and society		106(93.0)	41(100.0)	65(89.0)	0.07
Improvement of physician–patient relationships		83(72.8)	37(90.2)	46(63.0)	0.002
Improvement of the clinical staff's specialized knowledge		83(72.8)	35(85.4)	48(65.8)	0.02
Increased income of clinical staffs		29(25.4)	10(24.4)	19(26.0)	0.85
**Disadvantages of CR**					
Increased medical risk		55(48.2)	15(36.6)	40(54.8)	0.06
Greatly increased workloads		59(51.8)	23(56.1)	36(49.3)	0.49
Clinical staff's wages not greatly increased		62(54.4)	27(65.9)	35(47.9)	0.07
**Attitudes toward CR (whether supportive of CR)**					
Resident physicians		105(92.1)	39(95.1)	66(90.4)	0.59
Senior physicians		108(94.7)	41(100.0)	67(91.8)	0.15
Nurses or therapists		104(91.2)	39(95.1)	65(89.0)	0.45
Head nurses or head therapists		109(95.6)	40(97.6)	69(94.5)	0.78
Department administrators		113(99.1)	41(100.0)	72(99.6)	1.00
Hospital administrators		108(94.7)	40(97.6)	68(93.2)	0.57

*Data are presented as number (percentage) unless otherwise indicated*.

*CR, cardiac rehabilitation*.

### Clinical Staff Factors Associated With Phase II CR Quality During Implementation and Development

There were differences in “Improvement of physician–patient relationships” and “Improvement of the clinical staff's specialized knowledge” between the two groups. There were no differences in attitudes toward CR among six types of clinical staff between the two groups (Table 2).

### Factors Associated With Phase II CR Quality During Development and Implementation

**Figure 5:**
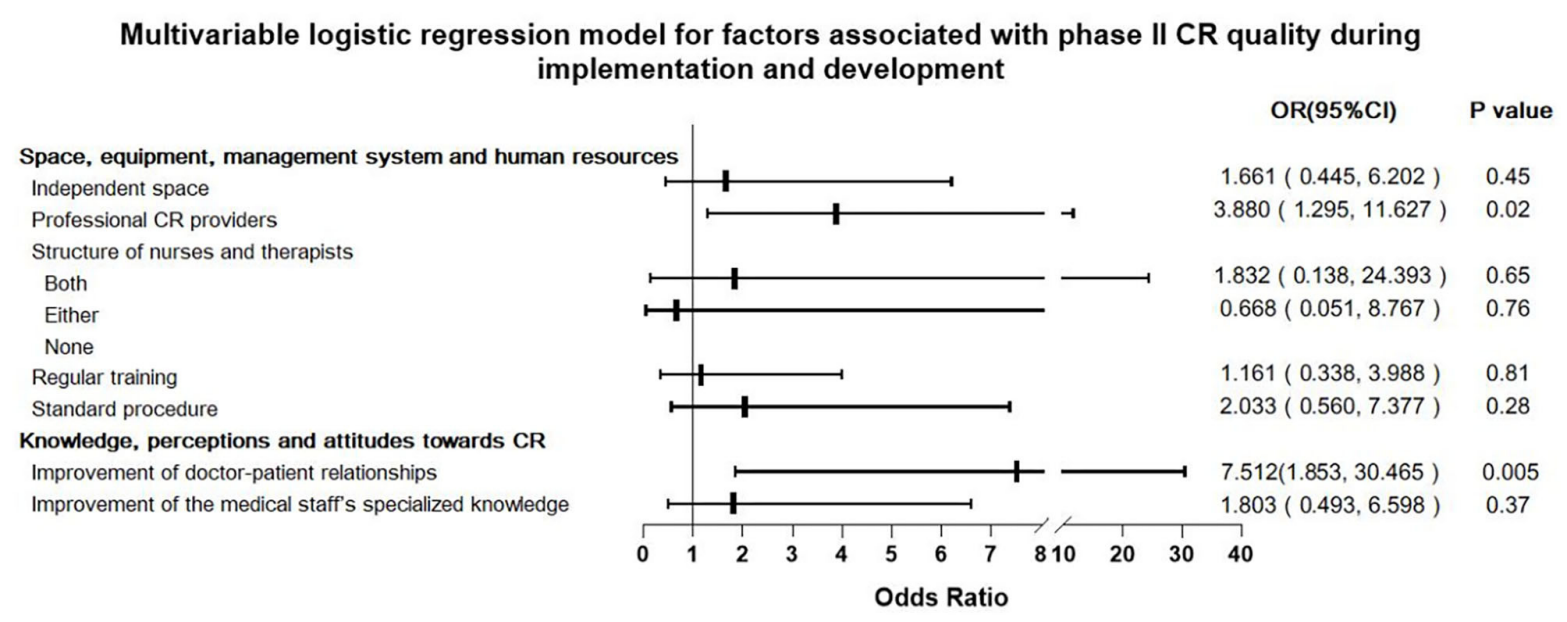
Multivariable logistic regression model for factors associated with phase II cardiac rehabilitation (CR) quality during implementation and development.

Multivariable logistic regression showed that availability of “Professional CR providers” and staff perception that CR improves physician–patient relationships are two independent predictors for phase II CR quality; their OR (95% CI) values were 3.880 (1.295–11.627) and 7.512 (1.853, 30.465), respectively (Figure 5).

## Discussion

Phase II CR implementation continues to face many difficulties throughout the process of implementing and developing CR programs (15). This study investigated and analyzed the factors on the implementation and development of phase II CR and also was the first study to explore the factors on the quality of CR via a nationwide survey. The data showed that after clinical staff had been trained, venues had been prepared, and equipment had/soon to be purchased, nearly one-third of these medical institutions had failed in developing a CR program. Moreover, of the remaining two-thirds of the institutions, 64% of them were unsuccessful in carrying out a high-quality program. This study showed that leadership support from hospital administrators, support from resident physicians, and staff perception in CR increasing medical risk were all independently associated with phase II CR implementation and development (Figure 3). The availability of “professional CR providers” and staff perceptions of CR improving physician–patient relationships were associated with CR quality during implementation and development (Figure 5). The quality of CR was unexpectedly not associated with medical system factors, and even though the department type was a significant indicator toward initial implementation and development success, thereafter, it was not associated with the quality of CR.

When analyzing the reasons behind this, institution leadership appears to play a key role. Hospital administrators are responsible for key decisions in hospitals (25), and as such, this study found that garnered support from these administrators was significantly associated with a successful implementation and development of a phase II CR program. Furthermore, support from resident physicians also appears to be associated with successful implementation and development. Patients are more receptive to the advice of the resident in-charge, so resident physicians play a key role in CR referral, thus affecting the effective implementation and development (8). This suggests that there is a need to gain the support of those in leadership roles within the hospital before the implementation and development of a phase II CR program.

Regarding clinical staff's perception and knowledge of CR, clinical staff recognized the beneficial effects of CR but cited the medical risk involved in treating patients as their main concern with implementing CR. Currently, research has shown that if strict procedures are followed, the risks involved in CR procedures are largely controllable (26–28), and physicians should not overestimate the risks (29). The medical personnel's awareness that CR can improve the relationship between physicians and patients will positively influence the quality of CR. This may be in part due to the current medical environment in China in that physician–patient relationships are not as positive as their international counterparts (30). If the medical staff believes that the doctor–patient relationship can be improved by CR, then they are more willing to promote CR development, thereby improving quality.

Additionally, the availability of professional CR providers in phase II CR programs also appears to influence the quality of CR. The professional level of medical personnel is impactful to the overall quality of CR programs. Because of this, the American Association of Cardiovascular and Pulmonary Rehabilitation (AACVPR) began to conduct Certified Cardiac Rehabilitation Professional (CCRP) certifications in 2014 and requires personnel to update their knowledge and skills every 3 years (31). In phase II, the constant improvement of professional skills and knowledge is indispensable, as well as the encouragement of clinical staff including physicians, nurses, and therapists, to actively participate in CR training to reach the primary qualification level (32). Therefore, at this stage, the Cardiac Rehabilitation associations around the world strive for government support and organize personnel training, attracting more professionals to join the CR program; this may be one of the key elements to accelerating development (32, 33).

Thought also must be placed on phase II CR location within a hospital. It is commonly acknowledged that carrying out CR in rehabilitation departments would be comparatively difficult compared to cardiology departments in China. Although rehabilitation departments are less likely to carry out CR programs than cardiology departments, once the program is set up, there is no difference in the quality of care delivered. Although the cardiology department may be able to control cardiovascular events risks very well, the rehabilitation department also has its advantages, such as better understanding the concept of rehabilitation and being accustomed to the technology used there (32, 34). Therefore, this suggests that the rehabilitation department is also able to develop a phase II CR program and deliver it at a high quality.

Medical system factors tend to play an important role in affecting the implementation and development of CR, mainly including characteristics of CR facilities and characteristics of the delivery systems (12). Generally, higher-level hospitals, public hospitals, general hospitals, teaching hospitals, and hospitals in large cities attract a greater number of patients due to their reputations and skillful clinical staff in China (23). Under the principle of performance appraisal, such hospitals have better financial and human resources to develop CR than the lower-level, private, specialized, non-affiliated hospitals and hospitals in small cities. However, these medical system factors did not affect the development and quality of CR. These results indicate that each hospital can achieve the basic requirements of CR and set up the CR program according to their own situation. There is a huge gap in medical insurance policies across China (35), and preceding studies have found that a lack of medical insurance is an obstacle to CR (16, 17). However, this study shows that both the medical insurance coverage and the reimbursement rate do not affect the quality of CR. Nevertheless, the promotion of insurance is conducive to further development of CR (36); also, good cost effectiveness brought by CR programs (37) will in turn promote the investment of medical insurance.

This study is not without limitations. Although the study covered most regions in China with varying levels of development and economies, variations between populations in other countries exist and will require further studies across different populations. In addition, patient factors were not included in the present study. Lastly, the survey was not analyzed for reliability and validity, but this survey had been fully discussed and revised by three cardiac rehabilitation experts and eight in-service training clinical staff; the final version of the survey was determined after three rounds of discussion. Such method designs ensured that the items and content of the survey were relatively reasonable.

In conclusion, before the implementation and development of phase II CR, support from those in leadership roles and resident physicians is required. Improving the awareness of the benefits and risks of CR through professional training can attract more relevant professionals and medical institutions to fully engage in CR programs. Regarding quality during implementation and development, CR programs should not be limited to specific departments or hospitals, as related departments in hospitals of all levels can carry out high-quality CR programs without viewing medical system factors as a major issue. Therefore, medical institutions that are planning to develop CR programs in the future may emphasize gaining support from those in leadership roles and training more medical personnel to improve the quality of CR.

## Data Availability Statement

The raw data supporting the conclusions of this article will be made available by the authors, without undue reservation.

## Ethics Statement

The studies involving human participants were reviewed and approved by Medical Ethics Committee of Xiangya Hospital of Central South University. Written informed consent was not provided because oral and digital informed consent was retrieved before the beginning of the survey.

## Author Contributions

SL had full access to all of the data in the study and takes responsibility for the integrity of the data and the accuracy of the data analysis. SL and XG contributed to the concept and design. SL, XG, WZ, JR-G, YD, YL, and LQ contributed to acquisition, analysis, or interpretation of the data. XG, JR-G, YL, and FZ contributed to drafting of the manuscript and contributed to statistical analysis. SL, XG, YL, and FZ contributed to critical revision of the manuscript for important intellectual content. All authors contributed to the article and approved the submitted version.

## Conflict of Interest

The authors declare that the research was conducted in the absence of any commercial or financial relationships that could be construed as a potential conflict of interest.
