# Supplementary material for: Successful Implementation and Development of a Phase II Cardiac Rehabilitation Program: A China-Wide Cross-Sectional Study Tracking In-service Training Clinical Staff
Source: Front Public Health. 2021 Mar 17;9:639273. doi: 10.3389/fpubh.2021.639273 (PMC8009984; doi:10.3389/fpubh.2021.639273)
Supplement: Supplementary file 1 [file Table_1.DOCX]

**Supplementary material**

**Methods and survey**

**MATERIALS AND METHODS**

**Design and procedure**

This study was cross-sectional in design. The investigation conforms with the principles outlined in the Declaration of Helsinki and was approved by the Medical Ethics Committee of Xiangya Hospital of Central South University (Ethics number: 202007212). The survey with the use of skip logic was made on the WenJuanXing Website (Professional survey production website, <https://www.wjx.cn/>). In-service training clinical staff trained in CR center of Xiangya Hospital were contacted through telephone or messages. The online survey was sent via WeChat (a messaging/calling app) to participants with informed consent. Respondents were encouraged to finish all the items; if a response wasn’t received after the first two weeks, phone calls and messages reminders were sent with a maximum of five attempts. Respondents could fill in the survey only once with their unique WeChat ID and the survey was restricted to forward to others in case of repetition and ghostwriting. The staff were considered as non-responders if they didn’t response the survey after five phone calls and messages reminders. The survey was collected from July to September 2019.

**Sample**

Since January 2013, CR Center of Xiangya Hospital has been recruiting in-service training clinical staff from other institutions to engage in CR training programs. The in-service training clinical staff were mainly from medical institutions that intended to and were preparing to administer their own CR program. These included physicians, therapists and nurses experienced in related departments such as cardiology and rehabilitation. The training included: [1] theoretical knowledge of CR, [2] nutritional, physical activity and mental state assessment, [3] practice of cardiopulmonary exercise testing (CPET), [4] prescription and the carrying-out of protocols in medicine, exercise, nutrition, psychology and tobacco cessation, [5)]management of sports injury, [6] prevention and management of cardiovascular events in CR programs, [7] CR practice in the outpatient clinic and inpatient department, and [8] alternative CR. Before practicing CR clinical work, the in-service training clinical staff must pass the CR certificate exam. We accessed the list of in-service training clinical staff from directory of in-service training clinical staff in Xiangya Hospital.

Inclusion criteria of the sample: [1] In-service training clinical staff who completed the CR training; [2] 3-6 months training length; [3] Undertook training between January 2013 and December 2018; [4] Passed the CR certificate exam; [5] Offered accurate contact information.

**Measures**

The following steps were taken to design the survey(1): [1] Literature review (databases including MEDLINE, EMBASE, Google Scholar, CNKI, Wanfang Data) to identify studies reporting results of CR program surveys on a regional, national or greater basis. [2] Invited three cardiac rehabilitation specialists from three different hospitals to discuss with the research team and produce initial 29 items of the survey based on the literature. The chosen cardiac rehabilitation specialists should meet some standards, including more than 20 working years in cardiovascular medicine, more than 10 years of experience in CR, senior technical title, and been academic leaders of their CR program. [3] Invited eight in-service training clinical staff certificated by CR training program, seven trained in Xiangya, one trained in another authorized program (Guangdong Province Hospital CR center) to pilot the initial survey. Of these in-service training clinical staff, 4 were female, 4 physicians, 2 nurses and 2 therapists. [4] Carried out a 20-90 minutes interview with eight piloters and requested their suggestions for revision. [5] The experts determined the final version after three rounds of discussion. The final questionnaire was composed of 30 items which included four parts, as below.

**Characteristics of in-service training clinical staff and their affiliated institutions**

The presented characteristics of the in-service training staff were gender, age, academic degree, clinical title, job title, and department, while those of their affiliated institutions were level, funding type, general/specialized, location city-level and academic type.

**Knowledge, perceptions and attitudes towards CR**

The knowledge and perceptions of the surveyed in-service clinical staff of CR, and their affiliated hospitals clinical staff’s attitudes towards CR, including resident physicians, senior physicians, nurses or therapists, head nurses or head therapists, department administrators and hospital administrators were recorded.

**Availability and characteristics of CR programs**

The types of cardiovascular disease and risk factors, content, exercise assessment, aerobic capacity assessment, exercise training, nutrition assessment and counseling, stress management were recorded (2).

**Space, equipment and management system and human resources for CR**

These factors included the availability of a dedicated CR outpatient clinic, an independent space, professional CR providers and the demographic distribution of providers (nurses or therapists). Whether there was regular CR training and standard procedure for CR, insurance coverage and reimbursement rate was noted.

**Standards for the development and quality of phase II CR program**

**Standards for the development of phase II CR program**

Phase II CR programs that offered services to patients following an acute cardiac event or hospitalization were of interest. This included residential programs. The inclusion criteria were CR programs that offered: [1] Initial assessment or risk assessment/stratification, [2] structured exercise (supervised or not), and [3] at least one other strategy to control CV risk factors (3).

**Overall quality of CR**

Overall, 16 structure and process quality indicators from CR societies were assessed through the survey (3). Eight core components (structure indicators) were recorded, including initial assessment, risk assessment/stratification, exercise training, patient education, management of cardiovascular (CV) risk factors, nutrition counseling, stress management and tobacco cessation interventions. Eight risk factors (process indicators) interventions were recorded, including blood pressure, lipids, physical inactivity, poor diet, adiposity, tobacco use, glucose/HbA1c, and depression. International Council of Cardiovascular Prevention and Rehabilitation (ICCPR) had published an international council of cardiovascular prevention and rehabilitation consensus statement in 2016, which recommended low and middle-income countries should broadly implement CR programs and deliver all core CR components (4). Furthermore, authorized CR programs were requested to provide all of quality indicators in China (5, 6), most of which was also consistent with quality indicators for CR in developed countries (7-12). Therefore, CR programs providing 16 quality indicators were categorized as high-quality, otherwise lower-quality.

**Data analysis**

Data analysis was performed with SPSS (version 24 for Windows; IBM Corp, Armonk, New York, USA). Data is described in Frequency (percentage) or mean (SD). To explore the factors associated with CR development, all cases were divided into two groups (successful implementation and development institutions, failed implementation and development institutions). Furthermore, to explore the factors associated with CR quality during implementation and development, all cases of successful implementation and development institutions were divided into either a high-quality group or a lower-quality group (Figure 1). A chi-square test was used to compare group differences in category variables and an independent-sample t-test was used for quantitative variables. The factors with *P* <0.05 were chosen for the next multivariable logistic regression analysis to identify independent factors associated with the development and overall quality of CR, *P* <0.05 was considered statistically significant. The odds ratio (OR) and 95% confidence interval (95% CI) were also calculated.

**References**

1. Turk-Adawi KI, Terzic C, Bjarnason-Wehrens B, Grace SL. Cardiac rehabilitation in Canada and Arab countries: comparing availability and program characteristics. BMC Health Serv Res. 2015;15:521.

2. Cortes-Bergoderi M, Lopez-Jimenez F, Herdy AH, Zeballos C, Anchique C, Santibanez C, et al. Availability and characteristics of cardiovascular rehabilitation programs in South America. J Cardiopulm Rehabil Prev. 2013;33(1):33-41.

3. Supervia M, Turk-Adawi K, Lopez-Jimenez F, Pesah E, Ding R, Britto RR, et al. Nature of Cardiac Rehabilitation Around the Globe. EClinicalMedicine. 2019;13:46-56.

4. Grace SL, Turk-Adawi KI, Contractor A, Atrey A, Campbell NR, Derman W, et al. Cardiac Rehabilitation Delivery Model for Low-Resource Settings: An International Council of Cardiovascular Prevention and Rehabilitation Consensus Statement. Prog Cardiovasc Dis. 2016;59(3):303-22.

5. Initiative CDQ. Evaluation criteria for national standardized cardiac rehabilitation centre. 2020 [Available from: <https://www.cdqi.org.cn/CDAssessment/crc_standard.aspx?to=crc>.

6. National Center for Cardiovascular diseases CMA. Cardiovascular disease quality initiative 2020 [Available from: <https://www.cdqi.org.cn/CDAssessment/crc_evaluation.aspx?to=crc>.

7. Grace SL, Poirier P, Norris CM, Oakes GH, Somanader DS, Suskin N. Pan-Canadian development of cardiac rehabilitation and secondary prevention quality indicators. The Canadian journal of cardiology. 2014;30(8):945-8.

8. Ohtera S, Kanazawa N, Ozasa N, Ueshima K, Nakayama T. Proposal of quality indicators for cardiac rehabilitation after acute coronary syndrome in Japan: a modified Delphi method and practice test. BMJ Open. 2017;7(1):e013036.

9. Spertus JA, Eagle KA, Krumholz HM, Mitchell KR, Normand SL, American College of Cardiology/American Heart Association Task Force on Performance M. American College of Cardiology and American Heart Association methodology for the selection and creation of performance measures for quantifying the quality of cardiovascular care. J Am Coll Cardiol. 2005;45(7):1147-56.

10. Thomas RJ, Balady G, Banka G, Beckie TM, Chiu J, Gokak S, et al. 2018 ACC/AHA Clinical Performance and Quality Measures for Cardiac Rehabilitation: A Report of the American College of Cardiology/American Heart Association Task Force on Performance Measures. J Am Coll Cardiol. 2018;71(16):1814-37.

11. Thomas RJ, King M, Lui K, Oldridge N, Pina IL, Spertus J, et al. AACVPR/ACC/AHA 2007 performance measures on cardiac rehabilitation for referral to and delivery of cardiac rehabilitation/secondary prevention services endorsed by the American College of Chest Physicians, American College of Sports Medicine, American Physical Therapy Association, Canadian Association of Cardiac Rehabilitation, European Association for Cardiovascular Prevention and Rehabilitation, Inter-American Heart Foundation, National Association of Clinical Nurse Specialists, Preventive Cardiovascular Nurses Association, and the Society of Thoracic Surgeons. J Am Coll Cardiol. 2007;50(14):1400-33.

12. Zecchin R, Candelaria D, Ferry C, Ladak LA, McIvor D, Wilcox K, et al. Development of Quality Indicators for Cardiac Rehabilitation in Australia: A Modified Delphi Method and Pilot Test. Heart Lung Circ. 2019;28(11):1622-30.

**Survey to explore the factors in the implementation and development of phase II CR program**

**(English translation version)**

**Part I.**

**Characteristics of in-service training clinical staff**

**1. Sex**

| ○Male |
| --- |
| ○Female |

**2. Age (Years)**

_________________________________

**3. What’s your educational level?**

| ○Junior college |
| --- |
| ○Bachelor |
| ○Master and above |

**4. What’s your clinical title?**

| ○Physician |
| --- |
| ○Nurse |
| ○Therapist |

**5. What’s your job title?**

| ○Senior |
| --- |
| ○Sub-senior |
| ○Intermediate |
| ○Primary |

**6. What’s your working department?**

| ○Cardiology |
| --- |
| ○Rehabilitation  ○Others |

**Characteristics of in-service training clinical staff affiliated institutions**

**7.** **What’s your affiliated hospital’s level?**

| ○Primary |
| --- |
| ○Secondary |
| ○Tertiary |

**8. What’s your affiliated hospital’s funding type?**

| ○Public |
| --- |
| ○Private |

**9. What’s your affiliated hospital’s category?**

○General

○Specialized

**10. What’s your affiliated hospital’s academic type?**

| ○Teaching hospital |
| --- |
| ○Non-teaching hospital |

**11. Which city is your affiliated hospital located in? (Please specify)**

_________________________________

**Part II.**

**Knowledge, perceptions and attitudes towards CR**

**12.** **What are the main benefits and advantages of developing CR program in your department? (Multiple choices)**

| □Reduction in the morbidity and mortality of cardiovascular events |
| --- |
| □Improvement of quality of life |
| □Improvement of mental health |
| □Helping patients to return to home and society |
| □Improvement of physician-patient relationships |
| □Improvement of the clinical staff’s specialized knowledge |
| □Increased income of clinical staffs |

**13. What are the main disadvantages of developing CR program in your department? (Multiple choices)**

| □Increased medical risk |
| --- |
| □Greatly increased workloads |
| □Clinical staff’s wages not greatly increased |

**14. Are resident physicians supportive of CR in your department?**

| ○Yes |
| --- |
| ○No |

**15. Are senior physicians supportive of CR in your department?**

| ○Yes |
| --- |
| ○No |

**16. Are nurses or therapists supportive of CR in your department?**

| ○Yes |
| --- |
| ○No |

**17. Are head nurses or head therapists supportive of CR in your department?**

| ○Yes |
| --- |
| ○No |

**18. Are department administrators supportive of CR in your department?**

| ○Yes |
| --- |
| ○No |

**19. Are hospital administrators supportive of CR in your hospital?**

| ○Yes |
| --- |
| ○No |

**Part III.**

**Availability and characteristics of CR programs**

**20. Has your department developed CR program?**

| ○Yes |
| --- |
| ○No (If selected, skip to end). |

**21.** **What core components has your program developed now?**

| □initial assessment |
| --- |
| □risk assessment/stratification |
| □exercise training |
| □patient education |
| □management of CV risk factors |
| □nutrition counseling |
| □stress management |
| □tobacco cessation interventions |

**22. What risk factors has your program intervened now?**

| □blood pressure |
| --- |
| □lipids |
| □physical inactivity |
| □poor diet |
| □adiposity |
| □tobacco use |
| □glucose/HbA1c |
| □depression |

**Part IV.**

**Space, equipment and management regulations for CR**

**23.** **Has your program developed CR outpatient clinic?**

| ○Yes |
| --- |
| ○No |

**24. Is your program equipped with independent space?**

| ○Yes |
| --- |
| ○No |

**25.** **Is cardiac rehabilitation performed by professional CR providers in your department?**

| ○Yes |
| --- |
| ○No |

**26. What kind of professional CR providers are there in the CR team? (Multiple** **choices)**

| □Physicians |
| --- |
| □Nurses |
| □Therapists |

**27. Is there regular training for cardiac rehabilitation in your program?**

| ○Yes |
| --- |
| ○No |

**28. Is there standardized process for cardiac rehabilitation in your program?**

| ○Yes |
| --- |
| ○No |

**29. Is the cost of cardiac rehabilitation reimbursed by medical insurance in your** **program?**

| ○Yes |
| --- |
| ○No |

**30. What is the proportion of reimbursement for cardiac rehabilitation in your program?**

| ○75.0-100.0% |
| --- |
| ○50.0-74.9% |
| ○25.0-49.9% |
| ○0.0-24.9% |
